# Enabling STD-NMR fragment screening using stabilized native GPCR: A case study of adenosine receptor

**DOI:** 10.1038/s41598-018-26113-0

**Published:** 2018-05-25

**Authors:** Sébastien Igonet, Claire Raingeval, Erika Cecon, Maja Pučić-Baković, Gordan Lauc, Olivier Cala, Maciej Baranowski, Javier Perez, Ralf Jockers, Isabelle Krimm, Anass Jawhari

**Affiliations:** 1 CALIXAR, 60 avenue Rockefeller, 69008 Lyon, France; 2 GENOS, Borongajska cesta 83h, 10000 Zagreb, Croatia; 3 Université de Lyon, Institut des Sciences Analytiques, UMR 5280, CNRS, Université Lyon 1, ENS Lyon - 5, rue de la Doua, F-69100 Villeurbanne, France; 4 SWING Beamline, Synchrotron SOLEIL, L’Orme des Merisiers, BP48, Saint-Aubin, Gif-sur-Yvette, F-91192 France; 5 Inserm, U1016, Institut Cochin, Paris, France; 6 CNRS UMR 8104, Paris, France; 7 University Paris Descartes, Sorbonne Paris Cité, Paris, France

## Abstract

Structural studies of integral membrane proteins have been limited by the intrinsic conformational flexibility and the need to stabilize the proteins in solution. Stabilization by mutagenesis was very successful for structural biology of G protein-coupled receptors (GPCRs). However, it requires heavy protein engineering and may introduce structural deviations. Here we describe the use of specific calixarenes-based detergents for native GPCR stabilization. Wild type, full length human adenosine A_2A_ receptor was used to exemplify the approach. We could stabilize native, glycosylated, non-aggregated and homogenous A_2A_R that maintained its ligand binding capacity. The benefit of the preparation for fragment screening, using the Saturation-Transfer Difference nuclear magnetic resonance (STD-NMR) experiment is reported. The binding of the agonist adenosine and the antagonist caffeine were observed and competition experiments with CGS-21680 and ZM241385 were performed, demonstrating the feasibility of the STD-based fragment screening on the native A_2A_ receptor. Interestingly, adenosine was shown to bind a second binding site in the presence of the agonist CGS-21680 which corroborates published results obtained with molecular dynamics simulation. Fragment-like compounds identified using STD-NMR showed antagonistic effects on A_2A_R in the cAMP cellular assay. Taken together, our study shows that stabilization of native GPCRs represents an attractive approach for STD-based fragment screening and drug design.

## Introduction

G protein-coupled receptors (GPCR) represent one of the largest family of integral membrane proteins and constitute highly druggable targets^1–5^. GPCRs are known to undergo conformational changes upon ligand binding and signal transduction^6,7^. This conformational flexibility represents a bottleneck in protein production and crystallographic studies. To improve thermostability and conformational homogeneity, mutagenesis and protein fusion (such as T4 lysozyme) approaches have been developed for various GPCRs such as β1 adrenergic or adenosine A_2A_ receptors^8–10^. Co-expression of mini-protein G has also helped to stabilize A_2A_ receptor^11^. In addition to acting at the expression level, high-affinity ligands, lipids or lipid-like molecules can be added during membrane preparation, solubilization and/or purification to provide conformational or oligomeric stabilization^12–17^. Recently the adenosine A_1_ receptor was crystallized bound to a selective covalent antagonist, revealing therefore basis for subtype selectivity^18^. Another approach is also possible using antibody fragments (Fab) or nanobodies as crystallization chaperones to stabilize GPCRs and facilitate their crystallization^19–26^. Recently, *in situ* reconstitution of the adenosine A_2A_ receptor in spontaneously formed synthetic liposomes was described to allow microscopy visualization and radio-ligand binding^27^. Other artificial membrane generation tools were also described^28,29^.

Those technologic and methodologic advancements led to the resolution of 251 GPCR 3D structures deposited in the PDB^30–35^ and open new routes for drug discovery, including the fragment-based approach. The fragment-based method consists in screening weak-affinity small molecular-weight compounds against protein targets^36^. The technique is well established for soluble therapeutic targets, while few studies have been described for membrane proteins. Yet fragment screening could be particularly valuable in the case of GPCRs, for the development of allosteric modulators that can overcome the selectivity issue of orthosteric ligands^17,37^. Fragment screening has been performed using biological and biophysical assays, including SPR^38–41^ and NMR-based TINS technology^41,42^. Although these techniques have proved to be valuable, it is of high importance to develop orthogonal methods that enable robust identification and validation of fragment hits. Besides, both SPR and TINS approaches require the immobilisation of the receptors. By comparison to the Carr-Purcell-Meiboom-Gill sequence (CPMG) experiment used in the TINS technology, the so-called Saturation-Transfer Difference (STD) NMR experiment provides structural information through the discrimination of solvent exposed and buried hydrogens of the ligand bound to the receptor^43,44^. While it is acknowledged that the STD experiment is particularly efficient for fragment screening, this technique has not been successfully applied to purified GPCRs yet.

To allow drug design and fragment screening on wild-type GPCRs using STD experiments, we have developed a strategy using calixarene-based detergent to solubilize and stabilize native, full length and functional GPCRs. We have lately reported on a systematic solubilization method for membrane proteins that allows screening for suitable detergents^45–47^ and have described the use of novel calixarene-based detergents^46,48–50^. Here, we report our solubilization strategy using the adenosine A_2A_ receptor (A_2A_R) as a case study. A_2A_R belongs to the GPCR class-A family of membrane spanning proteins that is involved in the brain and immune system regulation^51^. A_2A_R is of high medical interest particularly in Parkinson disease^52^, and also in cancer immunology^53^. A_2A_R is also responsible for regulating blood flow to the cardiac muscle and is important in the regulation of glutamate and dopamine release^54,55^. The purified adenosine A_2A_ receptor shows enhanced thermostability, while its behavior in solution shows no sign of aggregation and the presence of homogenous populations of monomers and oligomers. Functionality was assessed by radioligand binding. The feasibility of the STD-based fragment screening is demonstrated with the observation of the binding of characterized agonist and antagonist compounds to A_2A_R. The STD-NMR screening results illustrate the advantage of the experiment to obtain rapid structural information and gain additional insight into the ligand interaction.

Taken together, this work shows that wild-type GPCRs can be screened to identify fragment hits using STD-NMR experiments, which will bring new information for drug discovery in particular for the identification of allosteric modulators. This work also changes the dogma that GPCRs are by default unstable proteins requiring stabilization by mutagenesis and describes a new strategy for the fragment screening of highly unstable and druggable targets.

## Results

### Functional expression of WT and full length A_2A_R

Full length and wild type A_2A_R was expressed in yeast (*Pichia pastoris*) and Sf9 (*Spodoptera frugiperda*) with a His-tag at the amino-terminal. As shown in Fig. S1A, A_2A_R expression in yeast was clone-dependent. A specific band was observed at ~40 kDa band using a specific A_2A_R antibody, mainly for clones 2 and 3. A sixty-nine hours induction gives higher yield than 21 hours for A_2A_R expression. Therefore, 69 hours induction and clone 2 were selected for further expression. Regarding insect cells expression, Sf9 insect cells exhibit better expression 48 and 72 hours post-infection (Fig. S1B**)**. Forty-eight hours post-infection time was used for further expression steps. To evaluate the localization of the expressed protein, we performed cell lysis and membrane fractionation. From both yeast and insect cells, two fractions corresponding to enriched internal membranes (15000 G centrifugation, 15 K) and plasma membranes (100000 G centrifugation, 100 K) were analyzed by Western blot (Fig. S1C–F). Fractionation of Sf9 cells shows that A_2A_R was expressed in the 100 K and 15 K fractions, similarly (Fig. S1D and S1F). This was not the case for yeast expression since most of A_2A_R was observed in the 15 K fraction (Fig. S1C and S1E). To verify if A_2A_R expression was functional, we performed radioligand binding using the well-characterized agonist CGS-21680. Saturation curves show a specific binding of ^3^H-CGS-21680 to all A_2A_R containing membranes (Fig. S1C–F and Table S9**)**. Extrapolated Kd was generated for each membrane fraction. Interestingly, similar Kd values of ~0.11 (±0.05) and 0.29 µM (±0.06) were observed for 15 K and 100 K Sf9 membranes fractions, respectively. In contrast, yeast enriched plasma membranes (100 K) showed a lower Kd (0.80 ± 0.26 µM) in comparison to enriched internal membranes (15 K) (0.31 ± 0.13 µM). Thus, A_2A_R expression was functional.

### Solubilization, purification and ligands binding

Since dodecylmaltoside (DDM) was already reported to successfully solubilize A_2A_R^31,33,56^ and given that calixarene based detergent (CALX) were recently described to have a positive impact on membrane proteins stability^46,47,49,57^, we performed co-solubilization with DDM/CHS in combination with CALX-R10 detergent. Figure S3A shows that it was easier to solubilize A_2A_R from Sf9 than from *P. pastoris* (compare lane 3 to 2). To assess A_2A_R N-glycome, total N-glycans attached to A_2A_R purified from both plasma and internal membranes of yeast and Sf9 were enzymatically released, fluorescently labeled, and analyzed by hydrophilic-interaction ultra-high-performance liquid chromatography with fluorescence detection (HILIC-UHPLC-FLD).

**Figure 1:**
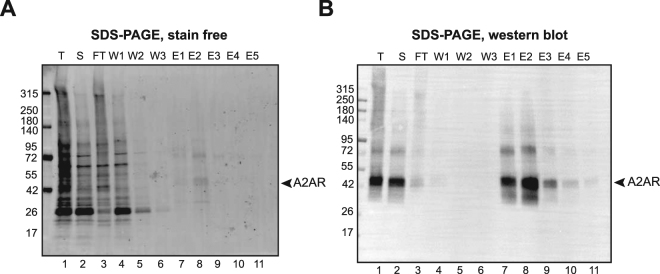
Purification of native A_2A_R. Talon affinity purification of A_2A_R from DDM/CHS/CALX-R10 solubilized total Sf9 membranes and analyzed by stain free SDS-PAGE (**A**) and western blot using antibody against A_2A_R (**B**).T, S, FT, W and E correspond to Total, Soluble, Flow Through, Wash and Elution fractions, respectively.

Figure S3 shows that glycosylation profile is preserved regardless of the localization of expressed protein (plasma vs internal membranes). This is true for A_2A_R expressed in *P. pastoris* and in Sf9. A_2A_R from *Pichia* was difficult to solubilize in comparison to Sf9. In addition to that, solubilization of A_2A_R from Sf9 offers the possibility to use both internal and plasma membranes for purification since they both have similar glycosylation pattern and similar ligand binding properties. We therefore combined Sf9 membranes (internal and plasma) for larger scale solubilization, purification and A_2A_R characterization. Good solubilization yields (~90%) were obtained for this detergent mixture as shown in Fig. 1B (compare lane 2 to 1). Most of A_2A_R could bind to the Talon-His column (Fig. 1B, compare lane 3 and 2) and elute specifically with a good purity (>90%) as shown in Fig. 1A (lane 8). Higher molecular weight gel migration of A_2A_R at ~80 kDa was observed. This corresponds most probably to SDS-resistant dimers since protein samples were not heated to avoid aggregation. This is commonly observed for membrane proteins. We then assessed radioligand binding of purified A_2A_R using ZM241385 (antagonist) and CGS-21680 (agonist). Table S9 shows obtained Kd values of 3.6 nM (±1.12) and 0.5 µM (±0.127 µM) for ^3^H-ZM241385 and ^3^H -CGS-21680, respectively. As comparative study, we have solubilized and purify A_2A_R using DDM/CHS and evaluated its ligand binding using ^3^H -CGS-21680 (Table S9). The obtained Kd was very similar to the one obtained using A_2A_R solubilized/purified in DDM/CHS/CALX-R10 (Table S9). This was also similar to Sf9 membrane bound forms and different from yeast membranes as shown in Fig. S1D, S1F and Table S9. This data shows that purified A_2A_R has maintained its ligand binding properties during the expression, solubilization and purification process.

### Behavior in solution and stability of purified native A_2A_R

**Figure 2:**
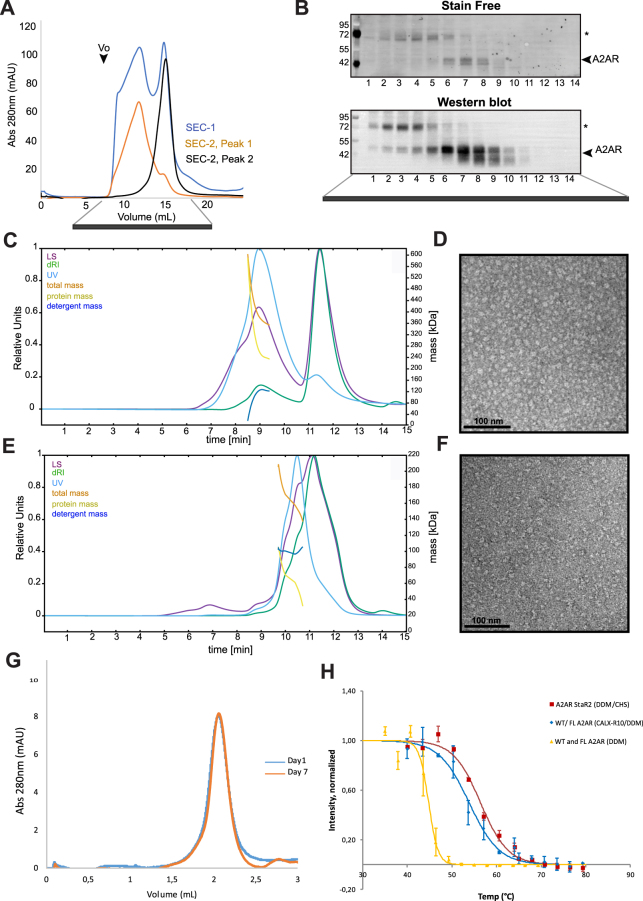
Behavior in solution and stability of purified native A_2A_R. (**A**) Gel filtration profile of A_2A_R showing two peaks (indicated as 1 and 2). Fractions corresponding to each peak were pooled, concentrated and used to run a second SEC as indicated by the red and black chromatograms. (**B**) Gel filtration fractions were analyzed by SDS-PAGE revealed by stain free (total protein) or western blot (A_2A_R only). Full length original gels are presented in Fig. S5. SEC-MALS analysis show the profile of peak 1 (**C**) and peak 2 (**E**). Light scattering (LS), differential refractive index (dRI), OD at 280 nm (y1 axes) and calculated masses (y2 axes) were plotted as a function of experiment’s time. OD, LS and dRI were rescaled to range from 0 to 100%, with 100% corresponding to maximum value of the curve. Negative stain image of A_2A_R fractions from Size exclusion chromatography peak 1 (**D**) and peak 2 (**F**). Scale bar correspond to 100 nm. Stability of solubilized/purified A_2A_R by Analytical Size exclusion chromatography (**G**). SEC was performed on affinity purified A_2A_R and gel filtrated protein (pool 2) after incubation 1 and 7 days at room temperature. SEC chromatograms were superimposed. Thermalshift assay (**H**). The assay was performed as described in methods on wild type and full length A_2A_R solubilized using two different conditions, CALX and reference corresponding to DDM/CHS/CALX-R10/ZM241385 and DDM/CHS, respectively. For comparison, A_2A_R StaR2, truncated (96 aminoacid c-terminal deletion) and mutated 8-point mutations solubilized and purified in DDM/CHS as described^78^ was also analyzed by thermalshift.

The next step was to assess the behavior of purified A_2A_ R in solution. To this end, we loaded his-tag affinity purified A_2A_R on a gel filtration column. Fig. 2A shows a typical profile of a non-aggregated protein since no peak was observed at the void volume Vo. Two peaks were noticed on size exclusion chromatography corresponding to two protein assemblies of different sizes. SDS-PAGE (stain free) and western blot analyses (Fig. 2B) show the existence of two populations of A_2A_R that migrate at ~80 and ~40 kDa and are abundant in peak 1 and peak 2 fractions, respectively. Similar profile in Size exclusion chromatography was also observed for A_2A_R StaR2 construct (96 aminoacid c-terminal truncation and 8-point mutations). The 80 kDa band corresponds most probably to SDS-resistant dimers. Peak 2 shows a second faster band consistent with a degradation product of A_2A_R. To investigate masses and thus oligomeric states of the protein, we performed a SEC-MALS experiment on both samples (peak 1 and 2, Fig. 2C, E respectively). The sample corresponding to peak 1 has a clearly defined protein peak at approximately 9.25 minutes, well separated from the free micelles peak at around 11.5 minutes. With dn/dc of the protein component set to 0.185 ml/g and dn/dc of the detergent set to 0.1618 ml/g, the mass of the protein component stabilizes around 240 ± 10 kDa. This corresponds approximatively to the theoretical mass of a A_2A_R pentamer, which is about 238 kDa. In peak 2, the protein and the free micelles are only partially separated. The protein UV maximum is at around 10.5 minutes and the free micelles dRI maximum at 11.2 minutes. Moreover, the shapes of all three spectra (UV, LS, dRI) suggest the presence of a second, smaller peak of protein with a maximum around 10 minutes. To evaluate the mass of the main peak of the protein, we limited the calculations only to data points between 9.7 and 10.7 min. Setting the dn/dc values as in sample/peak 1, we obtained the mass of the protein component strongly decreasing from 105 to 70 kDa between 9.7 and 10.0 minutes, then slightly stabilizing at 65 ± 5 kDa between 10.0 and 10.4 minutes, and finally strongly decreasing to 30 kDa until 10.7 minutes. The stable part thus displays a value slightly higher than that of a monomer. If we hypothesize that it corresponds to a mixture of monomer and dimer, then the proportions would be 63% monomer and 37% dimer. Figure 2D shows that A_2A_R particles of a size of ~10 by 10 nm could be observed for SEC peak 1 fractions. Much smaller particles were observed for SEC peak 2 fractions (Fig. 2F**)**. This is consistent with SEC-MALS finding that the first and second peaks correspond to higher and lower-order oligomers, respectively. To evaluate their ligand binding capacity, both peaks were analyzed by radioligand binding. Only A_2A_R from peak 2 showed convincing ligand binding (Table S9) in contrast to protein from peak 1. This strongly suggests that even if oligomeric A_2A_R was not obviously “aggregated”, it was not folded correctly enough to allow good ligand binding. We therefore focused on peak 2 for the next studies.

To evaluate the stability of the native GPCR, we submitted purified monomeric A_2A_R (peak 2 of a first gel filtration chromatography) to a second gel filtration run after 1 and 7 days incubation at room temperature. Figure 2G shows no decay of A_2A_R signal in size exclusion chromatography, arguing for good stability. To confirm A_2A_R stability we performed a western blot-based thermal shift assay. This assay relies on the assumption that unstable heated proteins will aggregate and after ultracentrifugation and western blot the band intensity corresponding to the protein will decay proportionally to its instability^58^. The result shown in Fig. 2H indicates that using CALX-R10/DDM condition (in the presence of ZM241385), A_2A_R exhibits a Tm of ~55 °C. The same A_2A_R is less stable in DDM with a Tm of ~43 °C as previously reported^58^ and confirmed in Fig. 2H. As a comparative study, we have expressed A_2A_R StaR2, solubilized it using DDM/CHS as described^8,59^ and submitted it to the same thermal shift assay. Figure 2H shows a 4 °C higher stability of A_2A_R StaR2 in comparison to A_2A_R wild-type and full length solubilized using CALX-R10/DDM. This is relatively minor considering that StaR2 contains 8 points mutations and a 96 amino acids truncation in the C-terminus. Thus, we could stabilize native, glycosylated, non-aggregated and homogenous A_2A_R that maintained its ligand binding capacity.

### Binding investigation of antagonists and agonists on A_2A_R using STD-NMR

**Figure 3:**
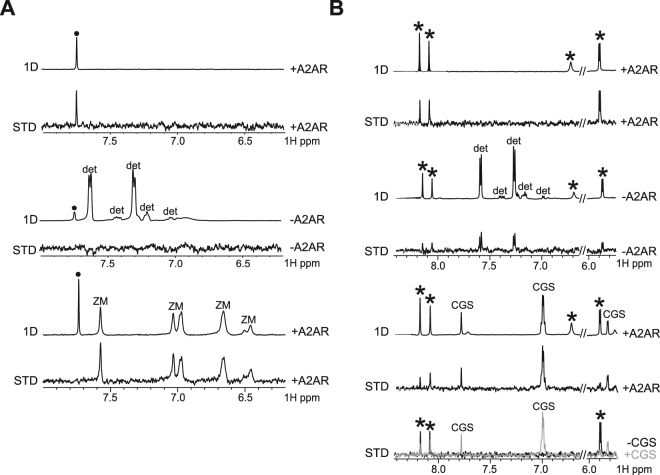
STD-NMR binding of A_2A_R to antagonists and agonists. 1D and STD NMR spectra of the caffeine antagonist bound to A_2A_R (**A**). The 1D and STD NMR spectra are also shown in the absence (middle) of the A_2A_ protein. NMR resonance of caffeine is indicated with a black dot and the aromatic compounds of the detergent buffer are labelled (det). The 1D and STD NMR spectra of caffeine are shown in the presence (bottom) of the ZM241385 compound. NMR resonances of the ZM241385 antagonist compound are labelled with the letters ZM. The STD binding signal of caffeine disappears in the presence of ZM241385. 1D and STD NMR spectra of the adenosine agonist bound to A_2A_R (**B**). The 1D and STD NMR spectra are also shown in the absence (middle) of the A_2A_ protein. NMR resonances of adenosine are indicated with a star and the aromatic compounds of the detergent buffer are labelled (det). The 1D and STD NMR spectra of adenosine are shown in the presence of the CGS-21680 agonist compound. NMR resonances of the CGS-21680 compound are labelled with the letters CGS. The STD binding signal of adenosine is weaker in the presence of CGS-21680. The intensities of the STD signals of adenosine in the presence and absence of CGS-21680 are superimposed (bottom) to illustrate the change in the STD intensities, particularly for the ribose resonance at 5.9 ppm.

STD experiment, which is a well-established NMR method for fragment screening against soluble therapeutic targets^44^, has not yet been used against purified GPCRs. Thus, we first wanted to demonstrate the feasibility of the approach through the binding investigation of known antagonists and agonists to A_2A_R. Fig. 3A shows the STD binding signal of caffeine bound to A_2A_R. By comparison, in the absence of the protein, the STD signal is considerably weaker, showing that the unspecific binding of caffeine to the micelles is insignificant. Competition experiment was performed by adding the A_2A_R antagonist ZM241385. As illustrated in Fig. 3A, the binding signal of caffeine disappears, while the binding signal of ZM241385 is observed. The STD experiment indicates that the caffeine binds to the same binding pocket as ZM241385, in agreement with the previously reported X-ray structures^30,31,60^. One expected advantage of the preparation of native A_2A_R is the possibility to observe the binding of agonists since the conformational flexibility of the receptor is not constrained in such a preparation. We have therefore investigated the binding of adenosine to A_2A_R. Figure 3B shows the STD spectrum of adenosine bound to A_2A_R. As for the caffeine, the STD signals are significantly weaker in the control experiment performed in the absence of the receptor. A competition experiment was achieved by adding the agonist compound CGS-21680. The STD intensities of adenosine decrease in the presence of CGS-21680, showing the competition between adenosine and CGS-21680 that both bind in the same binding pocket. Interestingly, adenosine still exhibits a significant STD signal in the presence of CGS-21680. This suggests that adenosine binds to another binding pocket when CGS-21680 is bound to A_2A_R. This finding will be further discussed in the discussion part. As illustrated in Fig. 3, the observation of the binding of agonists and antagonists to native A_2A_R as well as the competition experiments demonstrate that the fragment screening can be achieved using STD-NMR on the A_2A_R preparation.

### Fragment screening against A_2A_R using STD-NMR

**Figure 4:**
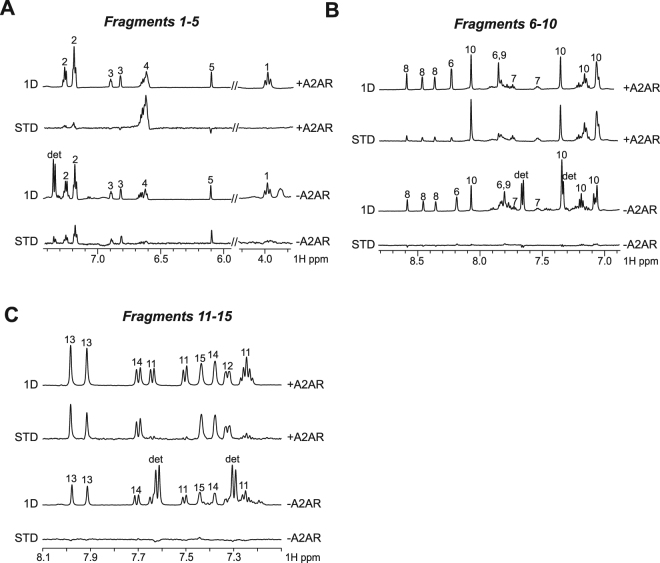
Fragment screening against native A_2A_R STD-NMR. The 1D and STD NMR spectra for fragments 1–5 (**A**), fragments 6–10 (**B**) and fragments 11 to 15 (**C**) are shown in the presence (top) and in the absence (bottom) of A_2A_R. The fragments bound to A_2A_R display STD signals, while the non-binders have no binding signal observed in the absence of A_2A_R, showing that the binding observed in the presence of native A_2A_R is specific.

We then performed fragment screening against A_2A_R using a hundred fragments. The molecules were screened into mixtures of 5 to 10, as typically done with soluble proteins. Fragments were then classified into three groups displaying strong binding, weak binding or no binding, depending on the intensity of the STD signals observed. Nineteen fragments (19%) were shown to exhibit significant STD intensities upon A_2A_R binding (Fig. S4). To further analyse the fragment screening results, cAMP cell-based assay was performed on ten fragments displaying either strong (fragments 4, 10, 12, 13, 14, 15) or weak binding (fragments 6, 7, 8 and 11) (Figs 4 and S7).

### Functional validation of fragments in the cAMP cell based assay

**Figure 5:**
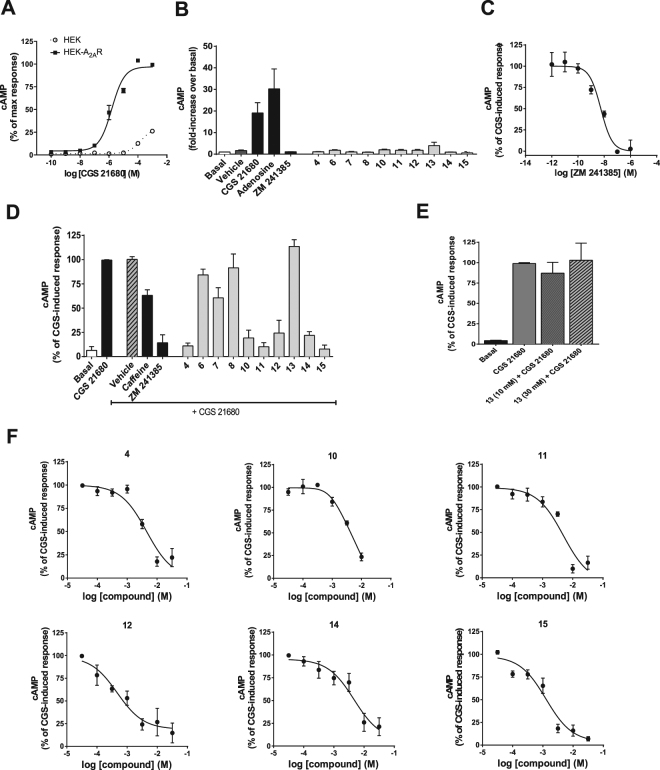
Functional validation of A_2A_R compounds on the cAMP signaling pathway. Concentration-response curves of CGS-21680-induced cAMP production in control HEK293 cells and in HEK293 cells stably expressing the A_2A_ receptor (HEK-A_2A_R) (**A**). Analysis of agonist effect of compounds on cAMP production (10 mM, 30 min). Vehicle: DMSO (1%); CGS-21680: reference agonist (1 µM); adenosine: reference agonist (1 µM); ZM241385: reference antagonist (1 µM) (**B**). Concentration-response curve of ZM241385 antagonist (15 min pre-incubation) on CGS-21680-induced (1 µM, 30 min) cAMP production **(C)**. Analysis of antagonist effect of compounds (10 mM, 15 min pre-incubation) on CGS-21680-induced (1 µM, 30 min) cAMP production. Vehicle: DMSO (1%); CGS-21680: reference agonist (1 µM); caffeine: reference antagonist (10 mM); ZM241385: reference antagonist (1 µM) (**D)**. Absence of antagonist effect of compound 13 (at 10 mM and 30 mM) on CGS-21680-induced cAMP production (**E**). Concentration-response curves of compounds, 4, 10, 11, 12, 14, 15 on CGS-21680-induced cAMP production (**F**). Data are expressed as mean ± S.E.M. of 3 to 6 independent experiments and normalized to either basal or CGS-21680-induced levels.

We then tested the capacity of the fragment binders shown in Fig. 4 to induce cAMP production in HEK293 cells stably expressing A_2A_R. CGS-21680 titration curves show that cAMP production is A_2A_R expression dependent (Fig. 5A). Non-transfected HEK293 cells were used as negative control and a small increase in cAMP production was observed associated to high concentrations of CGS-21680, probably due to the known presence of endogenous A_2A_R^61^. We then investigated the potential agonistic effect of the fragments displayed in Fig. 4. Stimulation of the A_2A_R cell line with different concentrations (10 µM, 100 µM, 1 mM and 10 mM) of each fragment for 30 minutes at room temperature had no effect on cAMP production even at the highest concentration of 10 mM as shown in Fig. 5B. CGS-21680 and adenosine served as positive controls and showed robust agonistic effects as expected. Accordingly, the well-established A_2A_R antagonist ZM241385 did not show any effect in this agonistic assay, while its efficiency in inhibiting CGS-21680-induced increase in cAMP production was confirmed (Fig. 5C). We then investigated the antagonistic effect of the fragments in the cAMP signaling assay. This test was performed by pre-incubating the A_2A_R cell line with the fragments (15 min, room temperature), followed by addition of the CGS-21680 agonist (30 min, room temperature). Fragments, 4, 10, 11, 12, 14 and 15 behave as full antagonists at 10 mM, whereas fragment 6, 7, 8 and 13 were without effect. Compound 13 remained inactive in this assay at concentrations up to 30 mM (Fig. 5E**)**. To further characterize the observed antagonistic effect, we generated full competition curves for fragments 4, 10, 11, 12, 14 and 15, which confirmed their antagonistic effect (Fig. 5F).

This result confirms the value of combining NMR-STD experiments and cell-based assays to discover functionally relevant fragments. Thus, using stabilized native A_2A_R, we could identify fragments with antagonistic effects on A_2A_R.

## Discussion

While dramatic progress has been achieved for structural and biophysical studies of membrane proteins such as GPCRs^37,62^, innovative approaches are still needed to discover new drugs targeting GPCRs. Significant efforts for the improvement of GPCRs stability has been made thanks to thermostabilization approaches by truncation, multiple alanine scan mutations and protein fusion. A_2A_R was significantly thermostabilized by mutating up to 8 residues at once and removing 96 amino-acids at the carboxy-terminal of the receptor^8^. A similar approach was successfully applied to other GPCR such as β-adrenergic receptor^9,63^. However, despite these approaches being very successful for structure determination, the modification of the protein sequence may restrict the repertoire of protein conformations existing in the native receptor and introduce a bias that may be misleading or limiting for structure-based drug discovery. Indeed, a recent NMR study demonstrates structural deviations of the fused receptor in the crystal due to the fusion^64^. In addition to that, even if the deletion of the c-terminus or the replacement of intra-cellular loops of GPCRs does not systematically impair ligand binding, these domains are crucial binding sites for interacting proteins important for receptor function^65^ and are thus likely to cause conformation deviations or restrictions from the receptor’s native state.

**Figure 6:**
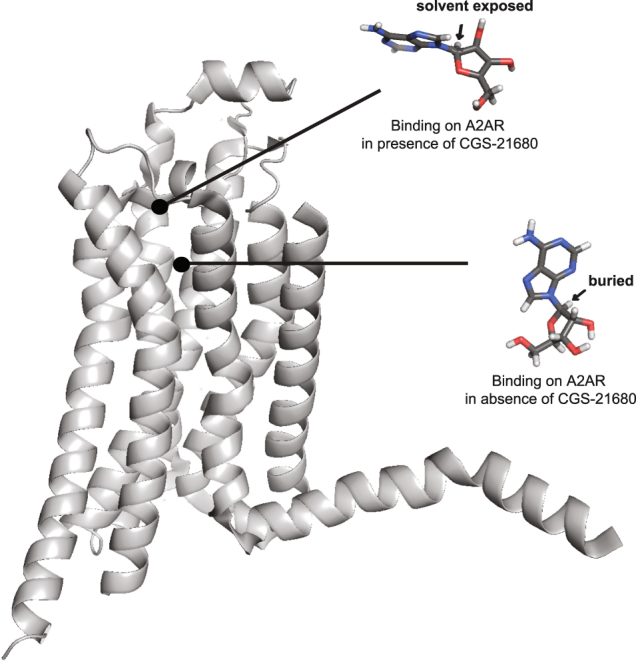
STD-NMR indicates a modification of the exposure to solvent for the ribose moiety of adenosine upon binding to A_2A_R, in the presence or in the absence of CGS-21680. The structure proposed for the adenosine with the buried ribose proton corresponds to the structure of the adenosine solved in complex with A_2A_R^33^ (PDB ID: 2YDO). The structure proposed for the adenosine with the solvent exposed ribose proton is inspired by the study of Sabbadin *et al*.^73^.

Here we report a stabilization approach for native, non-mutated GPCR. Here, we have used a specific calixarene-based detergent to solubilize and stabilize native, full length and functional A_2A_R. Native A_2A_R was stable for at least one week at 25 °C and showed a Tm of ~ 55 °C, corresponding to a significant stabilization shift in comparison to that previously reported for WT A_2A_R truncated at the C-terminus^8,58^ where a Tm of ~40 °C was measured. The native A_2A_R showed no sign of aggregation in SEC or EM in solution. Functionality was assessed by radioligand binding demonstrating binding of well-characterized agonist and antagonist compounds. This illustrates also the absence of conformational constraint since agonist and antagonist compounds were both able to bind to the receptor. This was not the case for StaR preparation that is not able to bind correctly to agonists such as CGS-21680 or NECA in comparison to the wild type protein^8,59^. The present work describes a natural alternative to systematic mutagenesis/fusion approaches and changes the dogma that GPCRs are unstable proteins requiring systematic stabilization by mutagenesis. The strategy described here is certainly not a time-consuming task in comparison to systematic scanning mutagenesis. This approach may be generalized across GPCRs and other highly challenging and druggable targets such as ion channels and transporters. NMR has been previously used to study the interaction of small molecules to GPCRs^66–71^. However, only the TINS technology was applied to screen fragments against GPCRs prepared in micelles and immobilized on a resin^41,42^. Here we aimed to use the STD method, which has the advantage to provide structural information through the discrimination of solvent-exposed hydrogens from buried hydrogens for the ligand bound to the receptor^43,44^. STD experiments recorded for the antagonist caffeine and the agonist adenosine showed that both types of ligands could be observed as binders with the native A_2A_R preparation. As shown in Fig. 3B, the STD intensities of adenosine bound to A_2A_R not only are weaker upon addition of the agonist CGS-21680, but the profile of the STD intensities are also modified. Notably, the STD signal of the proton of the adenosine ribose moiety at 5.9 ppm is considerably smaller when CGS-21680 binds A_2A_R. This indicates that the adenosine ribose moiety is buried in A_2A_R in the absence of CGS-21680, while it is solvent-exposed in the presence of CGS-21680 (Fig. 6). This observation corroborates with previous investigation of the binding mechanism of GPCR ligands using molecular dynamics simulation^72^, showing the presence of transient binding sites also called metastable binding sites or ligand-entry sites as potential allosteric sites^37^. In particular, a metastable binding site was proposed for adenosine bound to A_2A_R^73^. It was shown that adenosine could bind at the entrance of the orthosteric binding site, with the ribose oriented towards the entrance, solvent-exposed, in agreement with the NMR observation. These results show that the benefit of the STD-NMR experiment is to provide structural information for ligands bound to the receptor in the presence or absence of other compounds. This information will likely be of high interest for the discovery of allosteric binders. In the reported study, 19% of the fragments displayed significant binding on A_2A_R using STD-NMR. Comparison of NMR results with the cAMP cell-based assays achieved for 10 fragments showed that four fragments (6, 7, 8 and 13) displaying STD signals did not exhibit biological activity. It is acknowledged that fragment screening typically requires orthogonal techniques to identify and validate fragment hits, due to the weak affinity of such binders^74^. Therefore, it is not surprising to observe differences between the STD-based screening and the cAMP cell-based assay. While STD signals for fragments 6, 7 and 8 were classified as weak, fragment 13 displayed large STD signals upon binding to A_2A_R (Fig. 4C). The binding of compound 13 to A_2A_R was confirmed by testing the fragment alone (not in mixture) using STD (Fig. S8). In addition, STD-based competition experiment with the agonist CGS-21680 shows that fragment 13 binds in the orthosteric binding site of A_2A_R (Fig. S8). While the chemical structure of fragment 13 is similar to the adenine of adenosine, no conclusion can be drawn based on the cAMP cell-based assay only. It cannot be excluded that compound 13 may exhibit a pattern of agonism or even antagonism, as it is widely accepted that the classification of ligands in terms of their pharmacological properties is entirely dependent on the functional readout that is assayed^75,76^. Further investigation will be achieved for fragment 13, which is not the focus of this study. In conclusion, the current study describes the first stabilizing detergent/surfactant-based approach for native GPCR stabilization. Our goal in this study was to provide an alternative to systematic mutagenesis approach. Our preparation of A_2A_R could bind to well characterized agonists (adenosine and CGS-21680) and antagonists (caffeine and ZM241385). This suggests a full conformational space of the receptor. Also, the benefit of the STD-based fragment screening was discussed, showing that structural information for the binders can be inferred from the screening experiments. The reported approach represents an attractive alternative to the classical large-scale library compounds screening using cell-based assays.

## Methods

### Full length and wild type A_2A_R Expression

For insect cells expression, the full–length human A_2A_R was cloned into pOET1 transfer plasmid in frame with N-terminal hemagglutinin signal sequence, Strep-tag II and 8xHis tag, and baculovirus was produced according to the manufacturer’s protocol (flashback ULTRA™ system, Oxford Expression Technologies). *Sf9* insect cells were infected with baculovirus at a density of 1.5 × 10^6^ cells ml^−1^, using a MOI of 1, and grown at 28 °C for 64 hours in an orbital shaker. After 64 hours, cell pellets were collected, washed in Hepes buffer pH 7.4, 200 mM NaCl, 1x protease inhibitor cocktail (Sigma), then stored at −80 °C until use. For yeast expression, the full–length human A_2A_R was cloned into into the pPICα A expression vector (Thermo Fisher Scientific) in frame with the α-factor signal sequence, a Strep-tag II and a 8xHis tag, and linearized using the restriction enzyme DraI. The linearized vector was transformed into the *P. pastoris* strains KM71 and GS115 by using the Pichia EasyComp™ Transformation Kit (Thermo Fisher Scientific). Clone selection was performed by selecting recombinant His+ clones on MD agar plates (1.34% (w/v) yeast nitrogen base without amino acids, 2% (w/v) dextrose, 0.00004% (w/v) biotin, and 1.5% (w/v) agar). To select for multicopy transformants, His+ clones were grown on Zeocin-YPD agar plates (1% (w/v) yeast extract, 2% (w/v) peptone, 2% (w/v) dextrose, 2% (w/v) agar, and 0.1 or 0.025 mg/ml Zeocin). Representative clones exhibiting resistance to Zeocin were tested for recombinant protein production by Western-blotting. The selected transformants were stored as glycerol stocks at −80 °C. Single *P. pastoris* colonies from high expressing clones were selected on YPD plates containing 0.1 mg/ml Zeocin. Cells from a single colony were used to inoculate 300 ml of BMGY medium. The culture was grown overnight at 30 °C to an OD600 of 2–6. A total of 1.5 L of BMGY was inoculated with 300 ml of the starter culture and grown for 4 hr to an OD600 of 2. The cells were spun down at 4,000 g for 15 min, the cell pellet was washed with double distilled water, and then the cells were spun down. The cell pellet was resuspended in BMMY to an OD600 of 1. The culture was incubated for 20 hours at 20 °C with shaking at 150 rpm at 28 °C, and cell pellets were collected, washed in Hepes buffer pH 7.4, 200 mM NaCl, 1x protease inhibitor cocktail, then stored at −80 °C until use.

### Lysis and Membrane fractionation

Frozen cell pellets were thawed, resuspended in Hepes buffer pH 7.4, 200 mM NaCl, 1x protease inhibitor cocktail, and lysed by mechanical cell lysis. Cell lysis was performed on ice using a BeadBeater homogenizer with 0.1 mm diameter glass beads. Membrane fractionation was then carried out at 4 °C by sequential centrifugations. For both insect or yeast cells expressing A_2A_, 3 centrifugations were performed: 500 g for 5 min, 15000 g for 30 min, and 100000 g for 45 min. Membrane pellets were washed twice in buffer containing high salt (1 M NaCl) to remove membrane associated proteins. Membrane enriched pellets were resuspended in Hepes buffer pH 7.4, 200 mM NaCl, 1x protease inhibitor cocktail and glycerol 10%, quantified using the *Pierce Micro BCA* Protein *Assay Kit* (Thermo Scientific), flash-frozen and stored at −80 °C until use.

### Protein solubilization & purification

#### Protein solubilization

Proteins from internal or plasma membrane fractions were incubated for 2 h at 4 °C at a final concentration of 5 mg/ml in 50 mM Hepes buffer pH 7.4, 200 mM NaCl, 1x protease inhibitor cocktail, and with 0.155% CALX-R10 (10-fold the critical micelle concentration or CMC) in combination with 0.5% DDM and 0.06% CHS (57-fold the CMC). Extraction without detergent and with SDS served as negative and positive controls, respectively. After solubilization samples were centrifuged at 100000 g for 45 min at 4 °C and an aliquot of the total extract, the pellet and the supernatant from each solubilization condition was analyzed by SDS-PAGE and western-blot.

#### His-tag affinity chromatography

The soluble protein fraction was loaded onto a TALON column equilibrated with 50 mM Hepes buffer pH 7.4, 200 mM NaCl, 0.05% DDM and 0.006% CHS. After 2 h incubation at 4 °C, resin was washed with 12 Column Volumes (CV) of Wash Buffer containing 50 mM Hepes buffer pH 7.4, 200 mM NaCl, 0.05% DDM and 0.006% CHS, 20 mM Imidazole. Target protein was eluted with 4 CV of washing buffer with 150 mM Imidazole. Samples of each fraction T, S, FT, W and E (corresponding to Total, Solubilized, Flow through, Wash and Elution, respectively) were analyzed by SDS-PAGE and western-blot.

#### Size exclusion chromatography

Affinity purified A_2A_R was concentrated using Centriprep contractors with a 50 K cut-off and loaded on a superdex 200 Increase 10/300 GL(GE-Healthcare) at 0.3 ml/min. Running buffer was 50 mM Hepes buffer pH 7.4, 200 mM NaCl, 0.05% DDM and 0.006% CHS. Elution was performed with 1.5 CV of running buffer and 150 µl-fractions were collected. Fractions were analyzed by SDS-PAGE and western-blot. To assess stability of A_2A_R, superdex 200 Increase 5/150 GL (3 ml) was used.

### SDS-PAGE and Western-blot

A_2A_R samples were denatured with 5x Laemmli buffer and incubated for 20 min at RT prior to analysis without heating to avoid aggregates formation. Proteins were separated by SDS-PAGE on a 4–15% acrylamide gel (4–15% Mini-PROTEAN® TGX Stain-Free™ Gel, *Bio-Rad*) and subsequently immobilized by electro-transfer to PVDF membrane. The immunodetection of A_2A_R was performed by using the SNAP i.d. system (*Millipore*) with either a primary A_2A_R antibody (mAb 7F6-G5-A2), *Santa Cruz Biotechnology*) or an anti-His HRP antibody. Quantification of the signal was performed using Image Lab 4.1 software from *Bio-Rad* to evaluate the extraction efficiency. SDS-PAGE were silver stained using Bio-Rad Dodeca Silver Stain Kit following supplier protocol or coomassie stained using the PageBlue™ protein staining solution.

### Clear Native-PAGE (CN-PAGE) and Western-blot

Non-denaturated proteins were separated by native-PAGE on a 4–15% acrylamide gel (4–15% Mini-PROTEAN^®^ TGX Stain-Free™ Gel, *Bio-Rad*) using 25 mM imidazole as anode buffer and 7.5 mM imidazole, 0.05% deoxycholate, 0.01% DDM as cathode buffer). Clear Native PAGE gels ran for 90 min at 200 V and 4 °C. Proteins were then immobilized by electro-transfer to PVDF membrane. The immunodetection of A_2A_R was performed by using the SNAP i.d. system (*Millipore*) with A_2A_R antibody.

### Protein quantification

Total protein concentrations in the plasma and the internal membrane fractions were determined with the micro BCA protein assay kit (*Pierce*) using the bovine serum albumin (BSA) as a standard.

### Negative staining electron microscopy

Protein samples at 40 µg/ml were adsorbed on 200 Mesh copper grids coated with formvar-C for 2 min at RT. Then grids with suspension were colored with 1% uranyl acetate for 1 min and observed on a transmission electron microscope (Jeol 1400 JEM, Tokyo, Japan) equipped with a Gatan camera (Orius 600) and Digital Micrograph Software.

### N-glycosylation analysis

Prior to deglycosylation membrane samples were desalted using ice-cold methanol (Merck, Darmstadt, Germany). Briefly, dried membrane samples were resuspended in 1 ml of ice-cold methanol and centrifuged for 15 min at 2200 g. The supernatant was carefully removed and the procedure was repeated. The remaining methanol was evaporated by drying down in the vacuum concentrator. Dried samples were dissolved in 30 μL of 1.33% SDS (w/v) and denatured by incubation at 65 °C for 10 minutes. The following steps of N-glycan release and fluorescent labelling were essentially as described previously^77^. After labelling, the free label and reducing agent were removed from the samples by hydrophilic interaction liquid chromatography solid-phase extraction (HILIC-SPE) using 0.2 μm GHP filter plates and ice-cold 96% acetonitrile. Fluorescently labelled N-glycans were separated by HILIC on a Waters Acquity ultra-performance liquid chromatography (UPLC) system (Milford, MA, USA) as described previously^77^. Briefly, labelled N-glycans were separated on a Waters BEH Glycan chromatography column, 150 × 2.1 mm i.d., 1.7 μm BEH particles, with 100 mM ammonium formate, pH 4.4, as solvent A and acetonitrile as solvent B. Separation method used linear gradient of 70–53% acetonitrile (v/v) at flow rate of 0.56 ml/min in a 23 minutes’ analytical run. Samples were maintained at 10 °C before injection, and the separation temperature was 25 °C. The identity of N-glycans separated by HILIC-UPLC was determined by matrix-assisted laser desorption/ionization time-of-flight mass spectrometry (MALDI-TOF-MS). Prior to MS analysis, fractions of each N-glycan chromatography peaks were collected, dried down in a vacuum concentrator and resuspended in 10 μL of ultrapure water. Aliquots of 2 μL were spotted onto a MTP AnchorChip 384 BC MALDI target (Bruker Daltronics, Bremen, Germany), mixed on plate with 1 µL of matrix solution (5 mg/ml 2,5-DHB, 1 mM NaOH in 50% acetonitrile) and left to dry by air. Recrystallization was performed by adding 0.2 µL of ethanol to each spot. Analyses were performed in positive-ion reflectron mode on an UltrafleXtreme MALDI-TOF-MS equipped with a Smartbeam-II laser and FlexControl 3.4 software Build 119 (Bruker Daltonics). The instrument was calibrated using a plasma N-glycome standard. A 25-kV acceleration voltage was applied after a 140-ns extraction delay. A mass window of m/z 1000 to 5000 with suppression up to m/z 900 was used for N-glycan samples. For each spectrum, 10 000 laser shots were accumulated at a laser frequency of 2000 Hz, using a complete sample random walk with 200 shots per raster spot.

### Ligands binding assay

#### Radioligand binding

This assay was performed at 4 °C in triplicate using 96 wells plate with U bottom. Protein at a final concentration of 24 µg/ml (1 µg–50 µL per well) was incubated at 4 °C in the presence of 3.6 µM ^3^H-CGS-21680 (0.6 µM final, 10 µL per well) (Perkin-Elmer NET1021250UC)+/− 1 mM of cold ligand (0.17 mM final) in binding buffer (50 mM Tris-HCl pH 7.4, 10 mM MgCl2, 0.5 mM EDTA) or ZM241385. After 2 h of incubation, 60 µL of 0,1% γ-globulin (prepared in wash buffer) and 120 µl of 25% PEG6000 (prepared in wash buffer) were added per well, mixed and incubated for 15 mins at RT.

Samples were then filtered using PEI-pre-coated GF/B plates (Perkin-Elmer, cat#6005177). Plates were washed 4 times with ice-cold wash buffer (50 mM Tris-HCl pH 7.4) and 25 µl of scintillation reagent was added per well. After 1 h of incubation, CPM detection was done using the Microbeta2 equipment (Perkin Elmer), applying 5 mins counting per well.

#### NMR binding

NMR experiments were acquired at 293 K on a Bruker AVIII 600 MHz spectrometer equipped with a cryoprobe and a SampleJet auto-sampler. NMR sample containing protein was recorded with 2 µM A_2A_R in a buffer consisting of 50 mM Hepes at pH 7.5, 200 mM NaCl and 0,05% DDM/0,005% CHS and 10% D_2_O. NMR experiment in the absence of the protein was recorded in a buffer consisting of 50 mM Hepes at pH 7.5, 200 mM NaCl, 0,05% DDM/0,005% CHS and 0,02% CALX-R10/0,002% CHS (1CMC). Saturation time was 2 secs per experiment. Fragment screening was performed in mixtures of 5 to 10 fragments at 600 µM for each fragment.

Competition experiments: NMR competition experiments were acquired at 293 K in the presence of 10% D2O on a Inova Agilent 600 MHz spectrometer equipped with a cryoprobe and an auto-sampler. NMR sample contained 1 µM of A_2A_R in a buffer consisting of 50 mM Hepes at pH 7.5, 200 mM NaCl and 0,05% DDM/0,005% CHS. Caffeine and adenosine were used at a final concentration of 600 µM; ZM241385 and CGS-21680 were solubilized in 100% DMSO-d_6_ and used at a final concentration of 360 µM. Saturation time for the STD was 2 secs.

### Thermostability assay

Membranes of A_2A_R (4 mg/ml total protein) were solubilized in different conditions (see solubilization method above) for 2 hours at 4 °C. Solubilized fractions were obtained after 100,000 g ultracentrifugation for 1 h at 4 °C. Solubilized fraction serves to make 50 μl aliquots to be submitted to one temperature each as part of a gradient of temperature ranging from 25 to 72 °C using PCR thermal cycler (PeqSTAR 2x gradient; Peqlab). Samples were then centrifuged 40 min at 20000 g and supernatants were analyzed by SDS-PAGE and western-blot using anti- A_2A_R antibody (7F6-G5-A2). The relative intensity of the target protein at each temperature was quantified on Western-blot using Image Lab software 4.1 from *Bio-rad*. Each condition was performed twice. Intensity was plotted as a function of the temperature, normalized and fit to the Boltzmann equation with the least square method using Solver Adds-in of Excel software. The method is described by^58^.

### SEC-MALS

SEC-MALS experiment were performed with a Phenomenex Yarra Sec. 3000-3, 300 × 4.6 mm column, using a setup of consecutive: Agilent 1260 Infinity UV detector with 1 μL G4212-60008 cartridge, Wyatt Dawn Heleos 18-angles light scattering detector and Wyatt Optilab T-rEX refractometer. Temperature was held constant at 20 °C. Flow rate was set to 0.3 ml/min. OD was measured at 280 nm. We used Wyatt’s ASTRA 6 to align the measurements from the detectors, take band-broadening into account and calculate masses of the components of the sample. The mass calculation requires the knowledge of dn/dc of each component. For the protein part, this was set to 0.185 ml/g and for the DDM/CHS part, we measured the value of 0.1618 ml/g. As we could not find a reference value of dn/dc for DDM/CHS mixture in the literature, we measured dn/dc of DDM and DDM/CHS. 1 g of saltless samples of DDM and CHS were dried overnight and weighted to measure the amount of water in the samples. Taking this correction into account, 1% stock solutions of DDM and DDM/CHS were prepared in the 50 mM, HEPES pH 7.4; 200 mM NaCl buffer. From this, we prepared the series dilutions to 1%, 0.8%, 0.6%, 0.4% and 0.2% concentrations. 4 ml of each concentration, followed by 1.5 ml of pure buffer was injected directly to refractometer, and the measurements were fit using ASTRA 6. We obtained dn/dc of DDM = 0.1378 ± 0.75% ml/g, which is well within range of values typically cited in the literature, and dn/dc of DDM/CHS = 0.1618 ± 0.19% ml/g. R^2^ of both fits was above 0.99.

### cAMP assay

Measurements of cAMP production were performed by Homogeneous Time-Resolved FRET (HTRF)-based assay using the commercially available cAMP-femto-Tb kit (Cisbio, Codolet, France), according to the manufacturer’s instructions. HEK293 cells stably expressing A_2A_ were distributed to 384-well plate and treated with the indicated compounds for 30 minutes at room temperature (test of agonistic effect). Alternatively, cells were pre-incubated with the compounds (15 minutes) followed by addition of the agonists CGS-21680 (1 μM, 30 minutes; test of antagonistic effect). After the incubation time, cells were lysed, incubated with the kit reagents (1 h, RT) and the measurements were done in the plate reader Tecan Infinite F500 (Tecan, Switzerland).

## Electronic supplementary material

Supplementary Figures
