# Supplementary material for: Enabling STD-NMR fragment screening using stabilized native GPCR: A case study of adenosine receptor
Source: Sci Rep. 2018 May 25;8:8142. doi: 10.1038/s41598-018-26113-0 (PMC5970182; doi:10.1038/s41598-018-26113-0)
Supplement: Supplementary file 1 — Supplementary Figures [file 41598_2018_26113_MOESM1_ESM.pdf]

TITLE: Enabling STD-NMR fragment screening using stabilized native GPCR: A case study of adenosine receptor

AUTHORS: Sébastien IGONET[1*], Claire RAINGEVAL[3*], Erika CECON[5,6,7*], Maja PUČIĆ-BAKOVIĆ[2], Gordan LAUC[2], Olivier CALA[3], Maciej BARANOWSKI[4], Javier PEREZ[4], Ralf JOCKERS[5,6,7], Isabelle KRIMM[3] & Anass JAWHARI[1¶]

SUPPLEMENTARY INFORMATION

Figure S1. Functional expression of the full-length and wild type $A_{2A}R$ in *Pichia pastoris* (**A**) and in *Sf9* insect cells (**B**). 3 clones of yeast were tested as indicated by #. Expression kinetic was performed after 21 and 69 hours for yeast and after 24, 48 and 72 hours for insect cells. The presence of $A_{2A}R$ was assessed by western blot using $A_{2A}R$ antibody. Radioligand binding on enriched internal **(C** and **D)** and plasma membranes fractions (**E** and **F**) from yeast (**C** and **E**) and Sf9 (**D** and **F**). Membrane fractions were analyzed by SDS-PAGE and Western blot. ZM241385 was used as radioligand. Kd were extrapolated from binding curves.15K and 100K correspond to internal (15000G fractionation) and plasma membranes (100 000G fractionation), respectively. Full length original gels are presented in Figure S5.

Table S2: N-glycan compositions of identified chromatographic peaks of $A_{2A}R$ (from *Sf9* or *pichia*) analyzed by mass spectrometry.

Figure S3: Characterization of $A_{2A}R$ glycans after purification from different enriched internal or plasma membranes from yeast or Sf9. Solubilization and talon affinity purification from each membrane fraction. T, P, S, FT, W and E correspond to Total, Pellet, Solubilized, Flow through, Wash and Imidazole elution fractions, respectively (**A**). HILIC-UPLC N-glycome profiles of $A_{2A}R$ purified from *P. pastoris* plasma membrane (**B**), *P. pastoris* internal membranes (**C**), Sf9 plasma membrane (**D**), and Sf9 internal membranes (**E**). Structures are depicted following the CFG notation. GU – glucose units. Full length original gels are presented in Figure S5.

Obtained glycan profiles allowed differentiation of eight and fourteen highest intensity glycan peaks released from $A_{2A}R$ purified from membranes of *P. pastoris* and Sf9, respectively (**Figure S3B-E**). Preliminary structural assignment of glycan peaks was based on their elution properties, performed exoglycosidase digestions (data not shown). N-glycan compositions were confirmed via matrix-assisted laser desorption/ionization time-of-flight mass spectrometry (MALDI-TOF-MS) following initial chromatographic separation (**Table S2**). Glycan profiling revealed three main results. First, glycan profiles of $A_{2A}R$ purified from plasma and internal membranes of *P. pastoris* were highly similar, both in glycan compositions and their abundance (**Figure S3B-C**). Second, glycan profiles of $A_{2A}R$ purified from plasma and internal membranes of Sf9 also showed a high degree of similarity (**Figure S3D-E**). Third, glycan profiles of $A_{2A}R$ purified from *P. pastoris* and Sf9 showed some differences. All the glycans released from $A_{2A}R$ expressed in *P. pastoris* were oligomannose-type structures having seven to thirteen mannose residues (M7-13) with M9 being the most abundant. Similarly, oligomannose structures dominated the glycan profiles of $A_{2A}R$ expressed in Sf9 but had less mannose residues in their composition (M3-10) and the most abundant was monofucosylated trimannosidic glycan (FM3). In addition, complex-type glycans in which antennae initiated by N-Acetylglucosamine (GlcNAc) residues are attached to the mannose (Man) residues of the core sugar sequence common to all N-glycans ($Man_3GlcNAc_2$) were also present.

<u>Figure S4:</u> **Chemical structures of fragments hits for $A_{2A}R$ identified from hundred fragments using STD-NMR.** Fragments are classified into two groups: the first group contains the fragments used for the biological assay, the second groups contain the other fragment hits.

<u>Figure S5:</u> **Initial gels used to prepare Figures by cropping only lanes of interest. A-** Gel corresponding to Figure S3A. **B-** Gel corresponding to Figure 2B. **C-** Gel corresponding to Figure S1 C, D, E and F.

<u>Figure S6:</u> **Gel filtration profile of $A_{2A}R$ StaR2 (purified in DDM/CHS). B-** Gel filtration fractions were analyzed by SDS-PAGE revealed by stain free (total protein) or western blot ($A_{2A}R$ only).

<u>Figure S7</u>: **Chemical structures of fragments used in the STD experiments reported in Figure 4.** Fragments are classified into three groups: the first group contains the fragments displaying large STD signals (strong binders), the second group corresponds to fragments displaying weak STD signals (weak binders) and the third groups are non-binders.

Figure S8: **STD-NMR investigation of fragment 13 binding to $A_{2A}R$.**

The 1D and STD NMR spectra for fragment 13 bound to $A_{2A}R$ are shown. Fragment 13 displays significant STD signals. The 1D and STD NMR spectra fragment 13 are shown in the presence of the CGS21680 agonist compound. NMR resonances of the CGS21680 compound are labelled with the letters CGS. The STD binding signal of fragment 13 is weaker in the presence of CGS21680.

Table S9: **Dissociation constant determined by radiobinding measurements for studied $A_{2A}R$ (membranes and purified proteins) samples**.

Figure S1

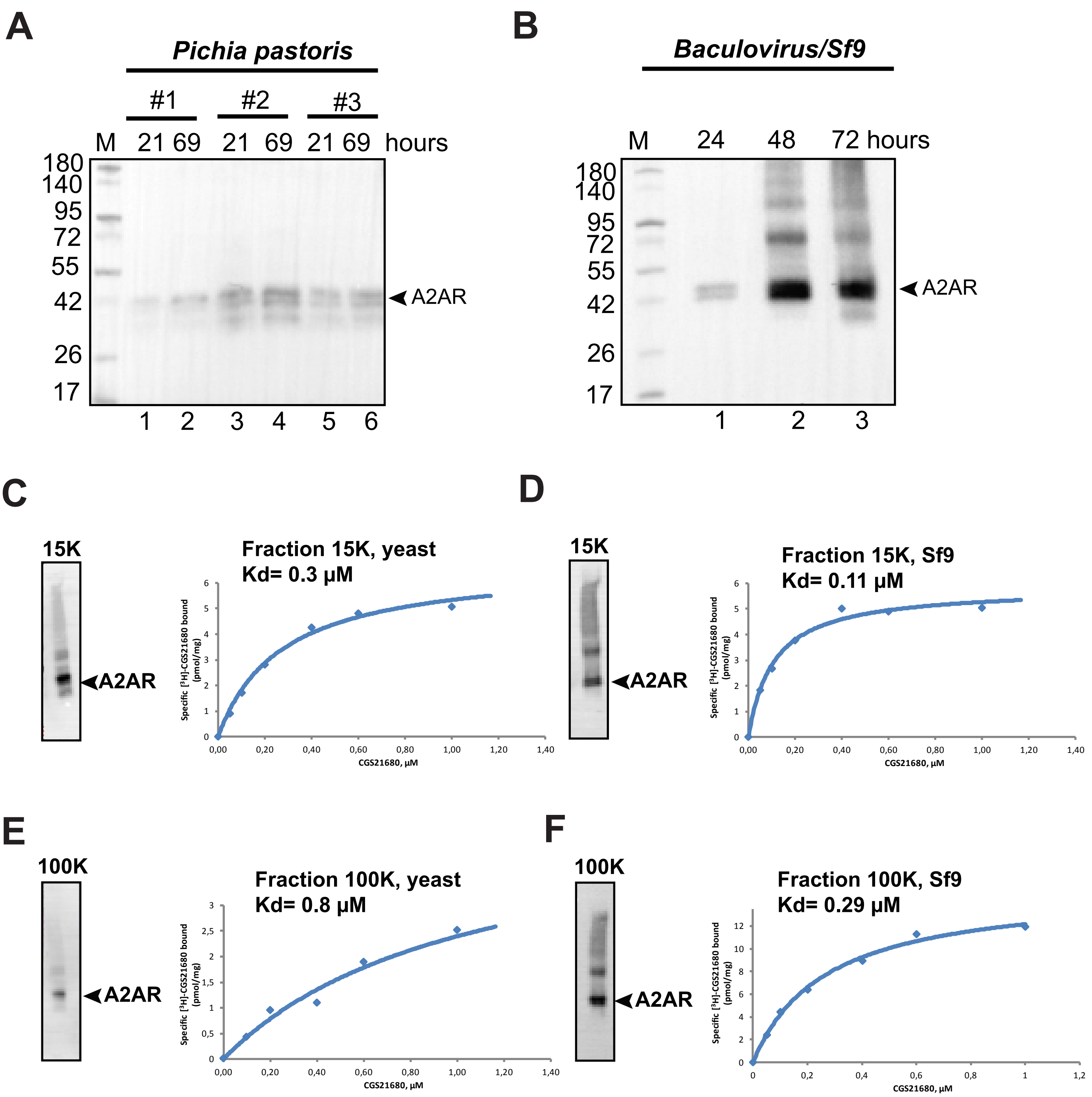

A
Pichia pastoris
#1
#2
#3
M
21 69 21 69 21 69 hours
180
140
95
72
55
42
26
17
A2AR
1 2 3 4 5 6
B
Baculovirus/Sf9
M 24 48 72 hours
180
140
95
72
55
42
26
17
A2AR
1 2 3
C
15K
A2AR
Fraction 15K, yeast
Kd= 0.3 µM
Specific [3H]-CGS21680 bound (pmol/mg)
CGS21680, µM
D
15K
A2AR
Fraction 15K, Sf9
Kd= 0.11 µM
Specific [3H]-CGS21680 bound (pmol/mg)
CGS21680, µM
E
100K
A2AR
Fraction 100K, yeast
Kd= 0.8 µM
Specific [3H]-CGS21680 bound (pmol/mg)
CGS21680, µM
F
100K
A2AR
Fraction 100K, Sf9
Kd= 0.29 µM
Specific [3H]-CGS21680 bound (pmol/mg)
CGS21680, µM

Table S2: N-glycan compositions of identified chromatographic peaks of A2AR

Sf9 A2A protein				
**Fraction**	**$[M+Na]^+$ m/z**	**Composition[a]**	**Structure[b]**	**Glycan scheme[c]**
**1**	1053.631	H3N2	M3	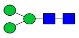
**2**	1199.539	H3N2F1	FM3	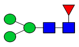
**3**	1402.684	H3N3F1	FA1	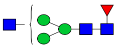
**4**	1605.730	H3N4F1	FA2	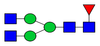
**5**	1377.639	H5N2	M5	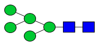
**6**	1767.838	H4N4F1	FA2G1	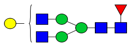
**7**	1539.690	H6N2	M6	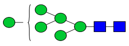
**8**	1685.885	H6N2F1	M6+F	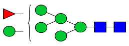
**9**	1701.894	H7N2	M7	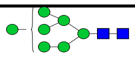
**10**	1864.05	H8N2	M8	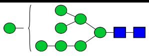
**11**	1864.041	H8N2	M8	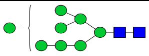
**12**	2025.891	H9N2	M9	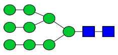
**13**	2025.832	H9N2	M9	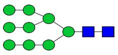
**14**	2187.806	H10N2	M10	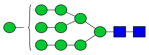

Sf9 P15000 membranes				
**Fraction**	**$[M+Na]^+$ m/z**	**Composition[a]**	**Structure[b]**	**Glycan scheme[c]**
**1**	1053.576	H3N2	M3	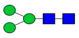
**2**	1199.432	H3N2F1	FM3	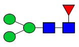
**3**	1402.771	H3N3F1	FA1	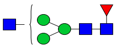
**4**	1605.744	H3N4F1	FA2	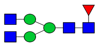
**5**	1377.322	H5N2	M5	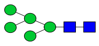
**6**	1767.415	H4N4F1	FA2G1	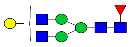
**7**	1539.293	H6N2	M6	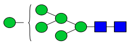
**8**	1685.437	H6N2F1	M6+F	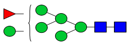
**9**	1685.865	H6N2F1	M6+F	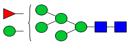
**10**	1701.640	H7N2	M7	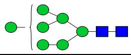
**11**	1863.296	H8N2	M8	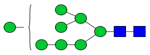
**12**	1863.326	H8N2	M8	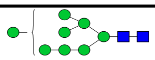
**13**	1863.672	H8N2	M8	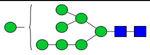

**14**	2025.556	H9N2	M9	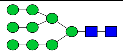
**15**	2025.779	H9N2	M9	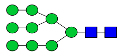
**16**	2187.810	H10N2	M10	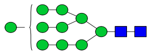
**17**	2349.642	H11N2	M11	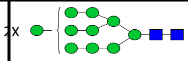
**18**	2511.432	H12N2	M12	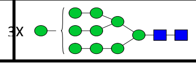

**Sf9 P100000 membrane**				
**Fraction**	**[M+Na]$^+$ m/z**	**Composition[a]**	**Structure[b]**	**Glycan scheme[c]**
**1**	1053.579	H3N2	M3	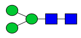
**2**	1199.385	H3N2F1	FM3	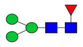
**3**	1402.571	H3N3F1	FA1	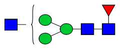
**4**	1605.529	H3N4F1	FA2	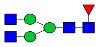
**5**	1377.679	H5N2	M5	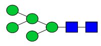
**6**	1767.861	H4N4F1	FA2G1	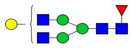
**7**	1539.629	H6N2	M6	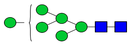
**8**	1685.726	H6N2F1	M6+F	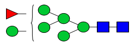
**9**	1685.930	H6N2F1	M6+F	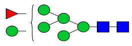
**10**	1701.603	H7N2	M7	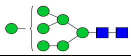
**11**	1863.790	H8N2	M8	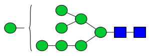
**12**	1863.835	H8N2	M8	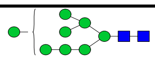
**13**	1863.777	H8N2	M8	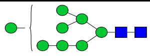
**14**	2025.732	H9N2	M9	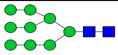
**15**	2025.752	H9N2	M9	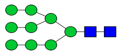
**16**	2187.482	H10N2	M10	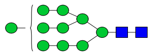
**17**	2349.863	H11N2	M11	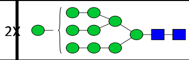
**18**	2512.015	H12N2	M12	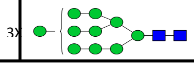

**Pichia pastoris A2A protein**				
**Fraction**	**[M+Na]$^+$ m/z**	**Composition[a]**	**Structure[b]**	**Glycan scheme[c]**
**1**	1701.658	H7N2	M7	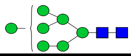
**2**	1863.585	H8N2	M8	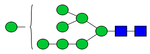
**3**	2025.679	H9N2	M9	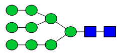
**4**	2187.596	H10N2	M10	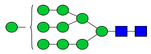

**5**	2187.672	H10N2	M10	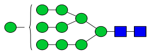
**6**	2349.892	H11N2	M11	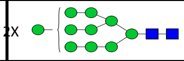
**7**	2511.736	H12N2	M12	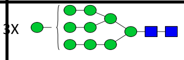
**8**	2673.493	H13N2	M13	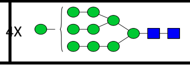

**Pichia pastoris P15000 membranes**				
**Fraction**	**[M+Na][+] m/z**	**Composition[a]**	**Structure[b]**	**Glycan scheme[c]**
**1**	1701.580	H7N2	M7	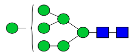
**2**	1863.451	H8N2	M8	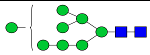
**3**	2025.631	H9N2	M9	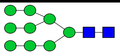
**4**	2187.517	H10N2	M10	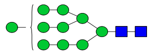
**5**	2187.452	H10N2	M10	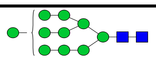
**6**	2349.596	H11N2	M11	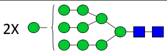
**7**	2511.356	H12N2	M12	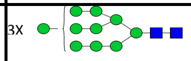
**8**	n.d.	H13N2	M13	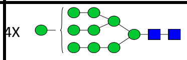
**9**	2835.192	H14N2	M14	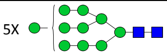

**Pichia pastoris P100000 membrane**				
**Fraction**	**[M+Na][+] m/z**	**Composition[a]**	**Structure[b]**	**Glycan scheme[c]**
**1**	1701.614	H7N2	M7	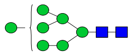
**2**	1863.592	H8N2	M8	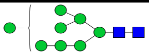
**3**	2025.587	H9N2	M9	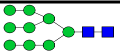
**4**	2187.386	H10N2	M10	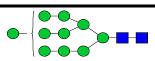
**5**	2187.475	H10N2	M10	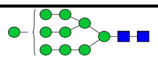
**6**	2349.459	H11N2	M11	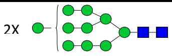
**7**	2511.469	H12N2	M12	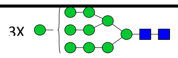
**8**	2673.280	H13N2	M13	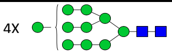
**9**	2835.600	H14N2	M14	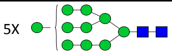

[a] H=hexose, N = N-acetylhexosamine, F = deoxyhexose (fucose)

[b] Abbreviations used: all N-glycans have a core sugar sequence consisting of two N-acetylglucosamines (GlcNAc) and three mannose residues; F at the start of abbreviation indicates a core fucose α1-6 linked to the inner GlcNAc; Mx, number (x) of mannoses on core GlcNAc; Ax, number (x) of antennas (GlcNAc) on trimannosyl core; Fx, number (x) of fucose linked α1-3 to antenna GlcNAc; Gx, number (x) of β1-4 linked galactoses on antenna.

[c] Symbols used: blue square = N-acetylglucosamine, green circle = mannose, red triangle = fucose, yellow circle = galactose

Figure S3

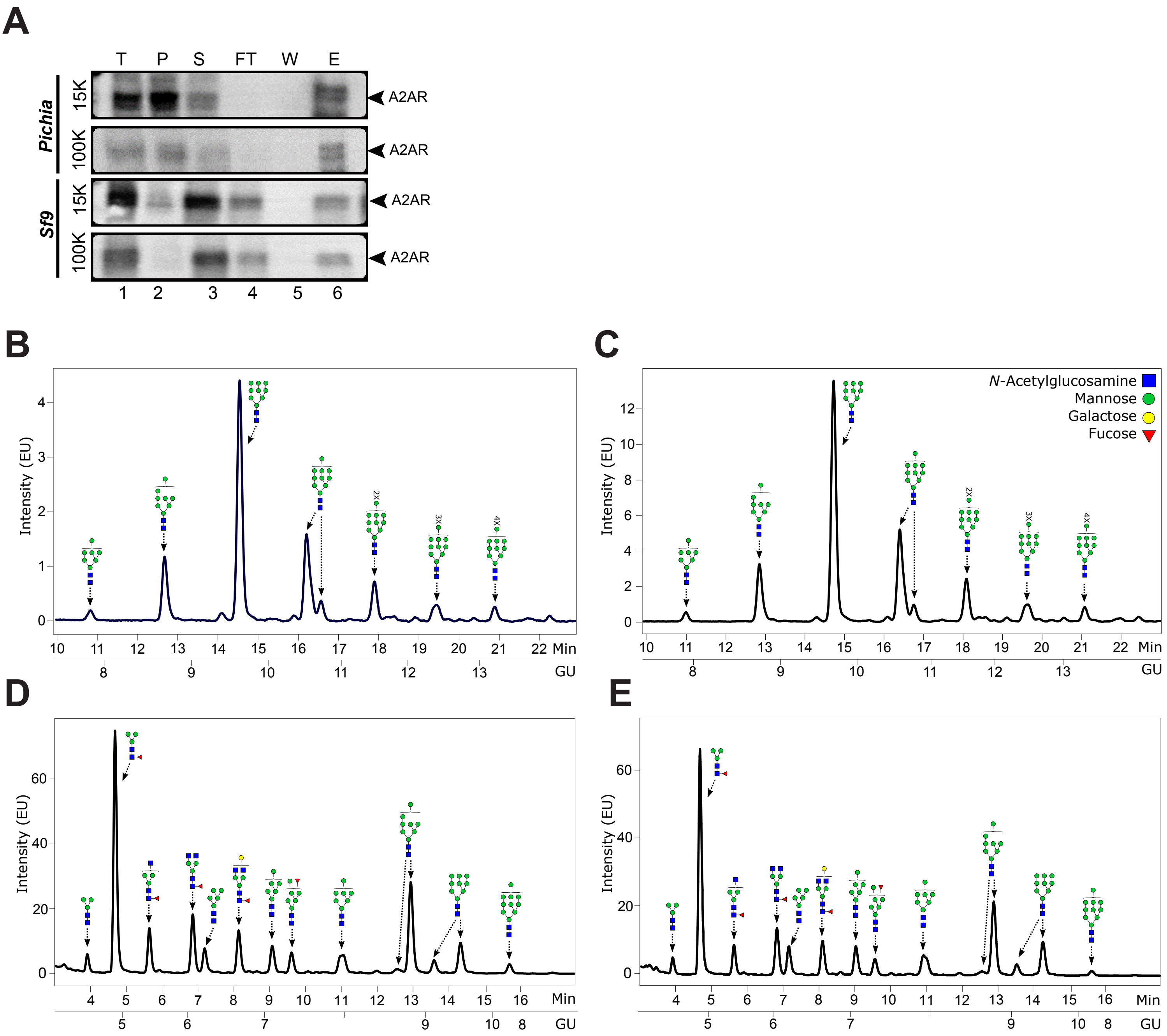
A
T P S FT W E
Pichia
Sf9
15K
100K
A2AR
1 2 3 4 5 6
B
C
D
E
N-Acetylglucosamine
Mannose
Galactose
Fucose
Intensity (EU)
Min
GU
2X
3X
4X

Figure S4

Fragments hits on A2AR using STD-NMR

Strong fragments hits selected for biological assay

4

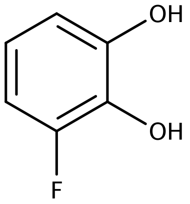

10

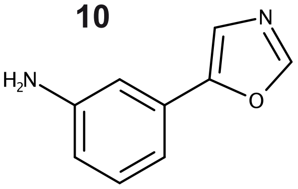

12

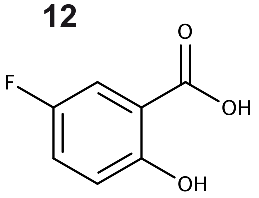

13

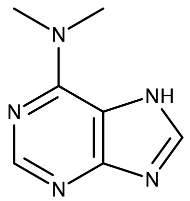

14

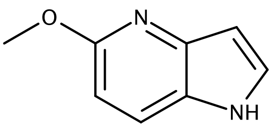

15

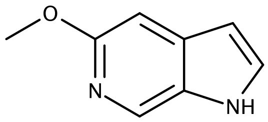

Others strong fragments hits from the screening

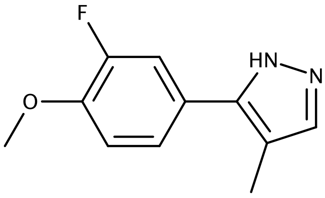

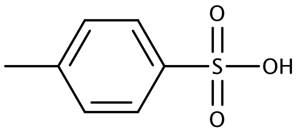

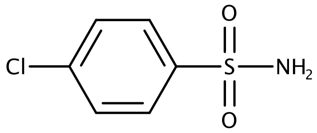

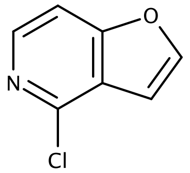

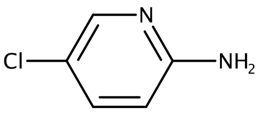

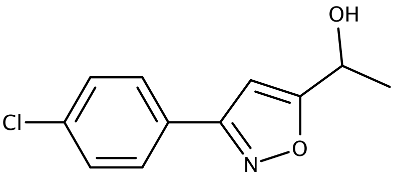

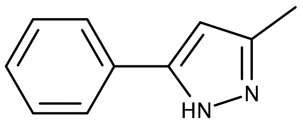

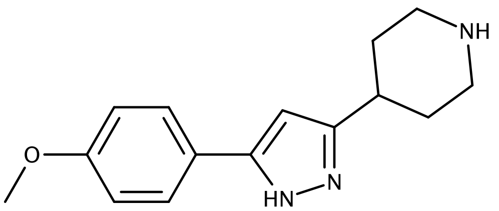

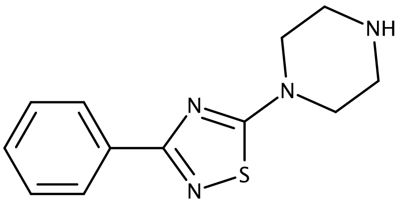

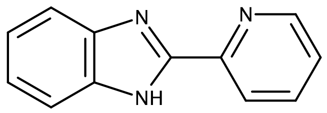

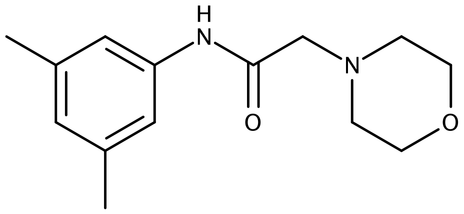

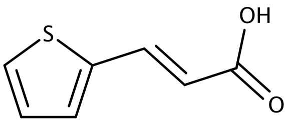

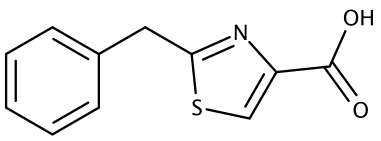

Figure S5

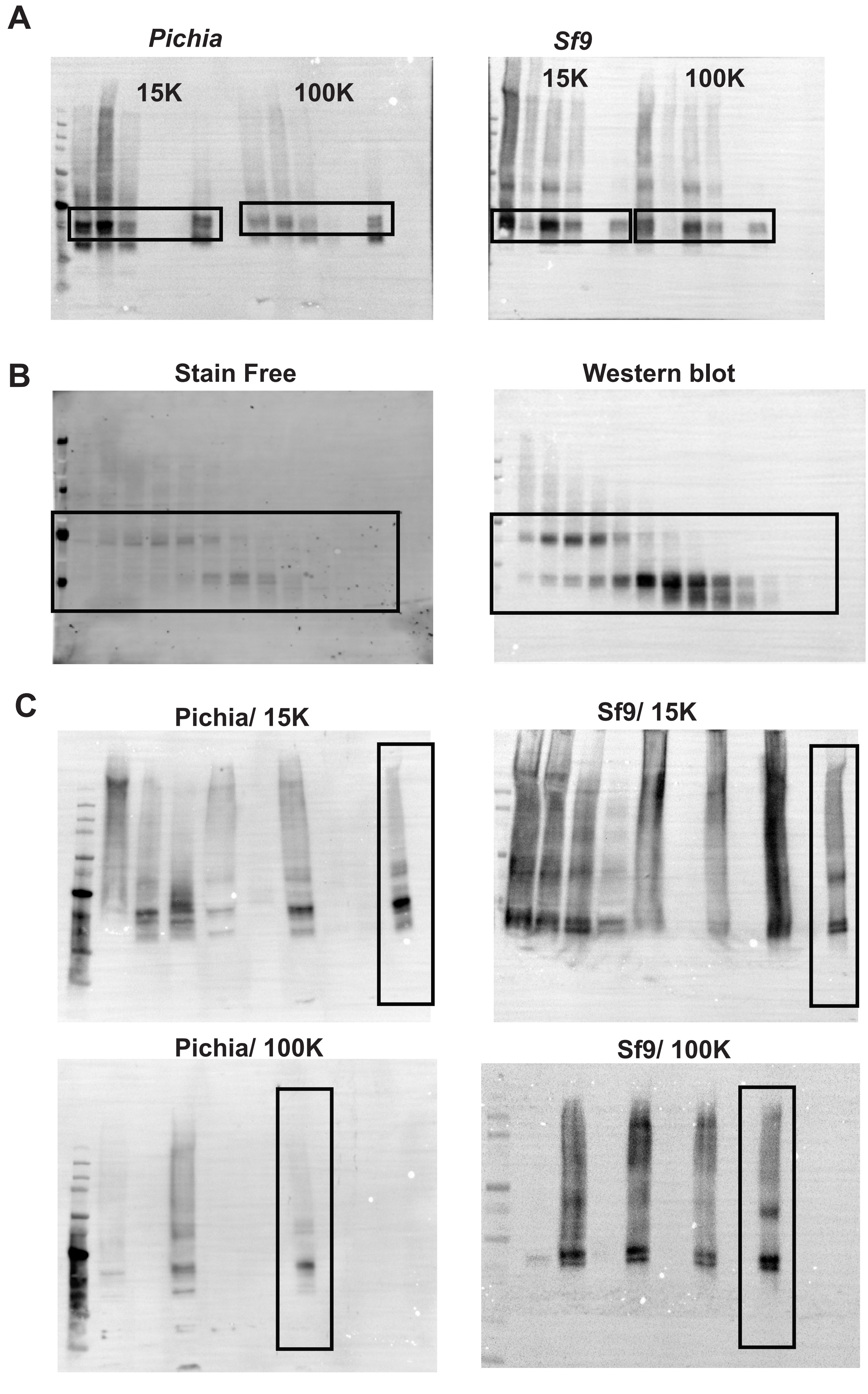
A
Pichia
15K
100K
Sf9
15K
100K
B
Stain Free
Western blot
C
Pichia/ 15K
Sf9/ 15K
Pichia/ 100K
Sf9/ 100K

Figure S6

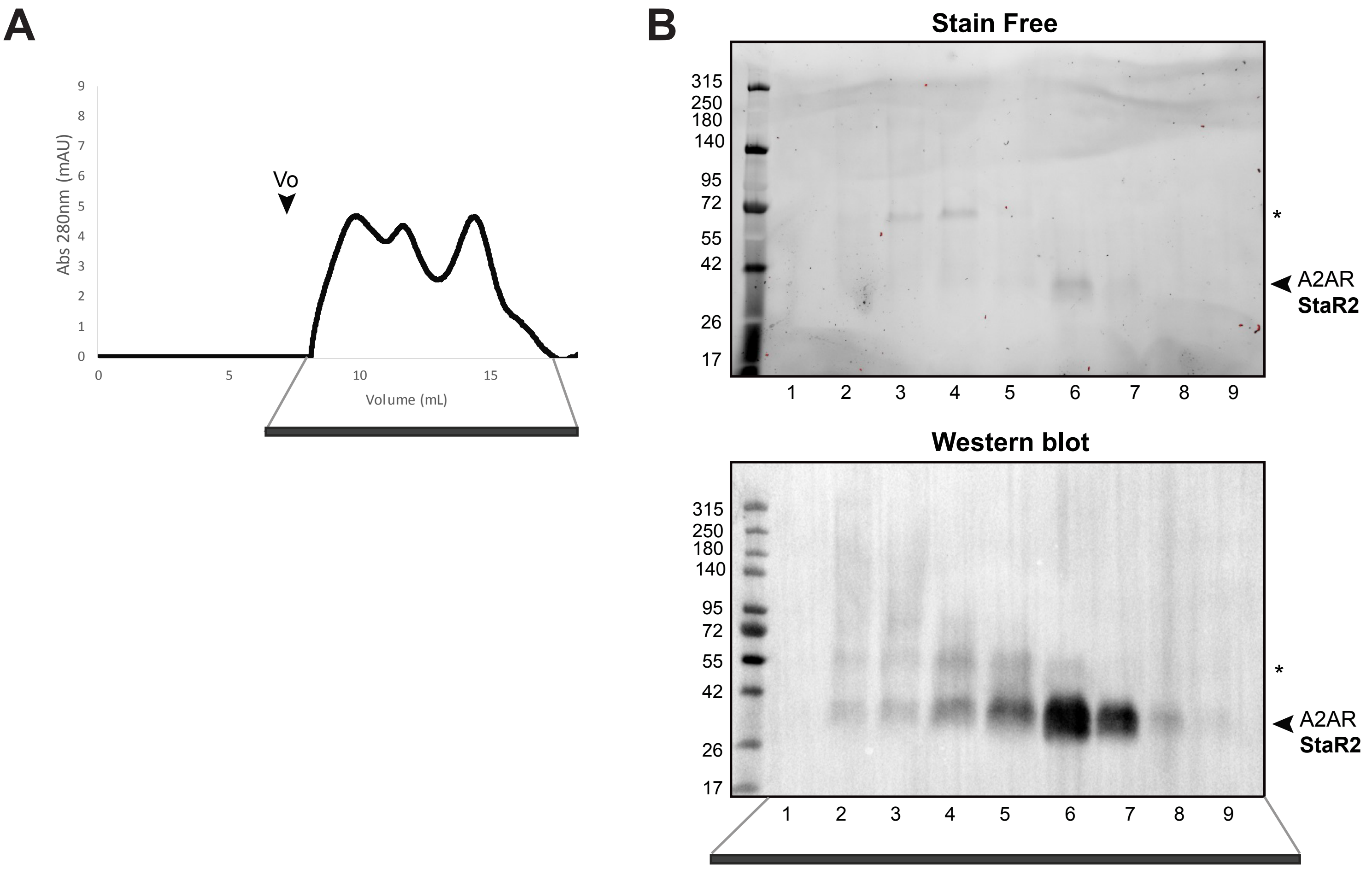
A
B
Vo
Abs 280nm (mAU)
Volume (mL)
0
1
2
3
4
5
6
7
8
9
0
5
10
15
Stain Free
Western blot
315
250
180
140
95
72
55
42
26
17
1 2 3 4 5 6 7 8 9
*
A2AR
StaR2

Figure S7

Strong NMR-STD binders

4

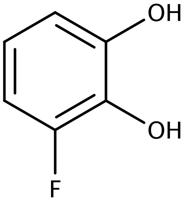

10

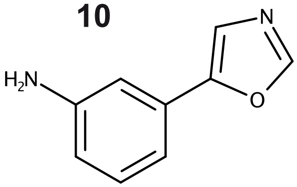

12

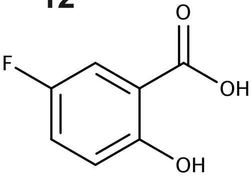

13

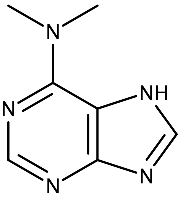

14

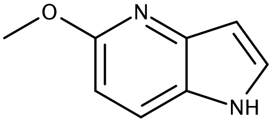

15

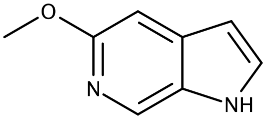

Weak NMR-STD binders

6

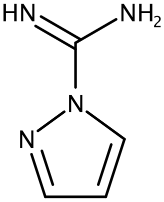

7

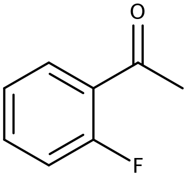

8

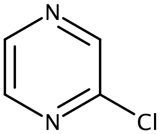

11

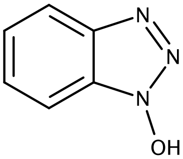

Non NMR-STD binders

1

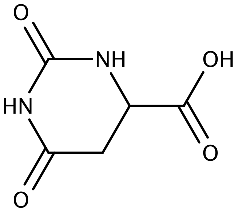

2

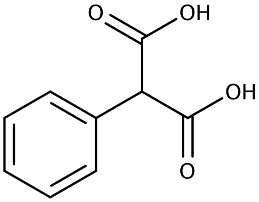

3

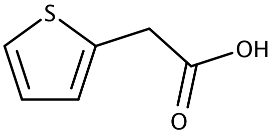

5

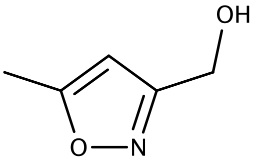

9

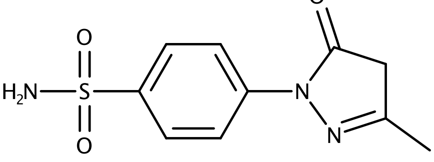

Figure S8

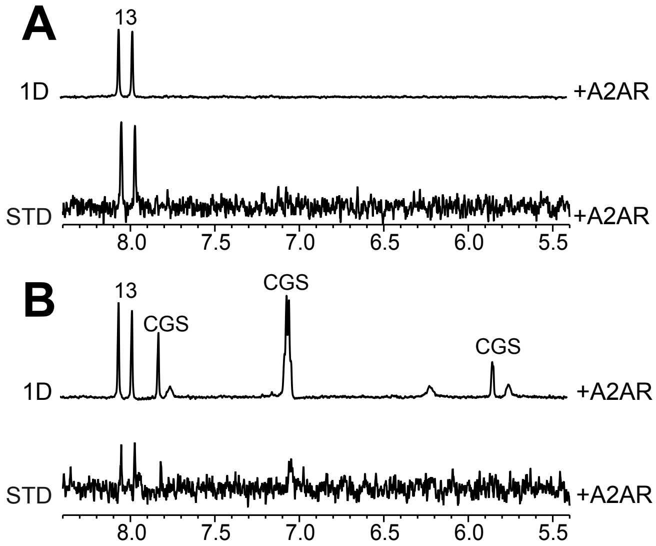
A
13
1D
+A2AR
STD
+A2AR
8.0 7.5 7.0 6.5 6.0 5.5
B
13
CGS
CGS
CGS
1D
+A2AR
STD
+A2AR
8.0 7.5 7.0 6.5 6.0 5.5

Samples (radioligand)	Kd (µM)
P. pastoris plasma A2A membranes (CGS 21680)	0.805 ± 0.262 µM
P. pastoris internal A2A membranes (CGS 21680)	0.307 ± 0.134 µM
Sf9 plasma A2A membranes (CGS 21680)	0.293 ± 0.06 µM
Sf9 internal A2A membranes (CGS 21680)	0.113 ± 0.055 µM
IMAC-purified A2A protein from DDM/CHS condition (CGS 21680)	0.444± 0.036 µM
IMAC-purified A2A protein from DDM/CHS/ CALX-R10 condition (CGS 21680)	0.5± 0.036 µM
IMAC-purified A2A protein DDM/CHS/CALX-R10 condition (ZM241385)	3.577 ± 1.116 nM
SEC-purified A2A protein Pool n°2 from DDM/CHS/CALX-R10 condition (CGS 21680)	0.1755± 0.041 µM
SEC-purified A2A protein Pool n°1 DDM/CHS/CALX-R10 condition (CGS 21680)	no binding detected

Table S9: Dissociation constant determined by radiobinding measurements for studied A2A samples.
